# The organization-level and physician-level factors associated with primary care physicians’ confidence in pandemic response: A multilevel study in China

**DOI:** 10.1371/journal.pone.0295570

**Published:** 2024-02-29

**Authors:** Haiming Chen, Tiange Xu, Rebecca Mitchell, Huiyun Yang, Zhongliang Zhou, Xiaolin Wei, Wenhua Wang

**Affiliations:** 1 Zhangjiagang Center for Disease Control and Prevention, Zhangjiagang, Suzhou, China; 2 School of Public Policy and Administration, Xi’an Jiaotong University, Xi’an, China; 3 Health and Wellbeing Research Unit (HoWRU), Macquarie Business School, Macquarie University, Sydney, Australia; 4 Dalla Lana School of Public Health, University of Toronto, Toronto, Canada; Dayeh University, TAIWAN

## Abstract

Primary care physicians (PCPs) suffered from heavy workloads and health problems during COVID-19 pandemics, and building their confidence in pandemic response has great potential to improve their well-being and work performance. We identified the organizational factors associated with their confidence in pandemic response and proposed potential management levers to guide primary care response for the pandemic. We conducted a cross-sectional survey with 224 PCPs working in 38 community health centers in China. Guided by self-efficacy theory, organization-level factors (organizational structure and organizational culture) and physician-level factors (job skill variety, perceived organizational support, work-family conflict, and professional fulfillment) were selected, and two-level ordinal logit models were built to examine their association with PCPs’ confidence in pandemic response. We found that hierarchical culture (OR = 3.51, *P*<0.05), perceived organizational support (OR = 2.36, *P*<0.05), job skill variety (OR = 1.86, *P*<0.05), and professional fulfillment (OR = 2.26, *P*<0.05) were positively associated with PCPs’ confidence in pandemic response. However, the influence of organization structure and work-family conflict seemed limited. The study not only increases our understanding of the influence of organizational context on PCPs’ pandemic response confidence, but also points out potential management levers for front-line primary care managers to enhance primary care pandemic response capacity.

## Introduction

As an unprecedented global crisis, every country has been making great efforts to better respond to the Coronavirus 2019 (COVID-19) pandemic during the past three years [1]. Global experiences show that high performing response countries generally have a resilient health system grounded in high quality primary care; while low performing response countries normally have fragmented health system and underfunded public health system with fewer mechanisms to link patients to primary care for routine care [2]. Recent analysis suggests that greater investment in risk communication and community engagement strategies as well as containing community transmission are the optimal strategies to improve pandemic preparedness and response for the next pandemic [3, 4]. These experiences and evidence point to the importance of building a strong primary health care system.

Strong primary health care-based health systems can respond quickly and effectively to pandemics [5]. Primary care physicians (PCPs) provide the foundation for a more resilient primary health care system, and are vital for public health emergency and everyday service delivery that ensure quality health services. Further, PCPs’ relationships with their communities may help address mistrust and stigma surrounding COVID-19 [5]. In China, PCPs refer to the physicians working in the primary health care organizations which consist of community health centers in urban China and township health centers and village clinics in rural China. PCPs are responsible for both individual-based clinical care and population-based health service provision, including COVID-19-relevant public health emergency services [6]. Because of the overwhelming workload without adequate support during the pandemic, these workers have substantially poorer health compared with general population [6–8]. For example, a recent meta-analysis showed that the prevalence rate of depression, anxiety, and insomnia among 33,062 frontline healthcare workers in COVID-19 affected countries reached 22.80%, 23.20%, and 38.90%, respectively, significantly higher than the general population [9, 10]. Poor mental health wellbeing and unsupportive working conditions also have significant negative implications for the provision of healthcare [11].

Confidence, often framed in terms of self-efficacy, is an individual’s judgment in his or her capabilities to perform behaviors required to attain designated levels of performance in a given context [12, 13]. Confidence strongly predicts physicians’ competence, behavior, and behavioral outcomes [14–16]. For example, greater confidence was significantly related to PCPs’ adherence to guidelines for the diagnosis and management of asthma [16]. Further, healthcare workers with a strong sense of confidence are more likely to accept challenging organizational goals and persist with associated tasks longer in the face of barriers than those with poor confidence [17]. Therefore, building PCPs’ confidence in pandemic response has great potential to improve their well-being and work performance.

Previous studies examining determinants of physicians’ confidence have been conducted in non-infectious disease care, such as pediatric palliative care, post-rape care, and musculoskeletal disorders care, with a focus on non-modifiable factors, such as physicians’ gender, prior training experiences, professional status, and work experiences [14, 18–20]. However, potentially modifiable factors within organizational domains, such as organizational structure and culture, the variety and meaningfulness of tasks, as well as work-related social support, are rarely studied [21, 22]. Therefore, this study aimed to examine the determinants of PCPs’ confidence in pandemic response from management perspective. The resulting data will increase our understanding of potential management levers that can build PCP’s confidence related to pandemic response and guide further primary care pandemic preparedness and response.

## Materials and methods

### Study design

We conducted a cross-sectional survey of 224 PCPs working in 38 community health centers (CHCs) in four large developed cities: Shanghai and Shenzhen in southern China, Tianjin and Jinan in northern China. In each city, the population size is nearly or over 10 million, and the GDP per capita is higher than the national average level. The primary care system in these cities, such as infrastructure building and primary care professionals, are relatively well established.

Considering the sample size requirements of multilevel study design as well as the feasibility of data collection during the pandemic, working with the local coordinators in the local health commissions, we used a convenience sampling approach to select a total of 38 CHCs of varying practice size and ownership in urban China: 10 in Shanghai, 14 in Shenzhen, 8 in Tianjin, and 6 in Jinan. At least 3 PCPs on duty in each CHC were invited to complete the physician survey on the day the researcher arrived [23]. Data were collected from November 2021 to May 2022 when China was implementing the zero-COVID policy, which had significant implications on the confidence of PCPs. Written informed consent was obtained from each participant before starting the anonymous survey using a self-administered questionnaire.

After the participants completed the questionnaire, our research assistant checked if there were any missing values to ensure the completeness of each questionnaire. Finally, 224 PCPs working in 38 CHCs were approached and all of them completed the physician survey, including data related to personal characteristics, professional attitudes, self-reported quality of care, and care confidence. The director of each CHC completed the primary care organization survey, including information related to organizational ownership, organizational size, medical equipment, organizational culture. Ethical approval for this study was obtained from the Ethics Committee at Xi’an Jiaotong University.

### Measures

#### PCPs’ confidence in pandemic response

We used the following item, “Do you have confidence in making your contribution to prevent and control COVID-19 pandemic in China?” to measure the PCPs’ confidence in pandemic response with a five-point Likert scale (1 =“not at all”, 5 = “extremely”). Activities relating to COVID-19 prevention and control included a wide range of activities, such as public health education, testing, screening, referring suspected or confirmed cases, monitoring and following-up community residents with potential disease contact, immunization, delivering services to patients with and without COVID-19.

#### Organization-level factors

Guided by the self-efficacy theory [12], two organization-level factors and four physician-level factors were selected as primary predictors. The two organization-level factors were organizational structure and organizational culture. One item, “The CHC emphasizes getting personnel to follow the formal procedures” was used to assess the organizational structure with a 7-point scale (1 = “strongly disagree”, 7 = “strongly agree”) [24]. The score (ranging from 1 to 7) of this item reflects the level of organizational hierarchy. The 20-item organizational culture survey based on Competing Values Framework (CVF) was used to measure the types of organizational culture in each CHC, which included four types of organization culture: group culture, developmental culture, hierarchical culture, and rational culture [25, 26]. Finally, a binary variable, hierarchical culture (emphasizing rules and regulations) or non-hierarchical culture, was created to measure organizational culture.

#### Physician-level factors

Four variables were used to measure PCPs’ professional characteristics, including job skill variety, perceived organizational support, work-family conflict, and professional fulfillment. Job skill variety was measured by one item “The job of being a physician in my CHC requires me to use different skills and abilities” with a five-point response option (1 = “strongly disagree”, 5 = “strongly agree”) [27]. A seven-item scale was used to measure perceived organizational support (1 = “strongly disagree”, 7 = “strongly agree”) [28]. The summed score of 4 items, e.g., “Your job reduces the effort you can give to activities at home”, was used to measure work-family conflict (1 = “strongly disagree”, 7 = “strongly agree”) [29]. Professional fulfillment was measured with a six-item scale with a five-point response option (1 = “not at all”, 5 = “completely”), e.g., “During the past two weeks, I feel happy at work.” [30].

#### Control variables

Sex, age, education level, years of working experience at the physician level, ownership type (government-owned CHC and hospital-owned CHC), organization size (the total number of primary care physicians in CHC), and information technology functional capacity at organizational level were selected as the control variables (see Table 1) [31]. To measure information technology functional capacity, PCPs were asked to rate the availability of 14 related items with “yes” or “no”, which were divided into three categories: “low” which equals two or fewer “yes”, “middle” with three to six “yes”, or “high” with seven to 14 “yes”[31]. All measure items had been used and validated in previous studies in China [32–38].

**Table 1:** Characteristics of 224 PCPs within 38 CHCs.

Characteristics	Total (n = 224)
**Physician-level**	
Sex, n (%)	
Male	90(40.18)
Female	134(59.82)
Education level, n (%)	
High school or below	9(4.02)
Undergraduate/college	179(79.91)
Master or above	36(16.07)
Age (years), n (%)	
<30	44 (19.64)
30–40	94 (41.96)
≥40	86 (38.39)
Years of working experience(years), n (%)	
<5	66 (29.46)
5–10	49 (21.88)
10–15	38 (16.96)
≥15	71 (31.70)
**Organization-level**	
Organizational ownership, n (%)	
Government-managed	142(63.39)
Public hospital-managed	82(36.61)
Information technology functional capacity, n (%)	
Low or middle level	55(24.55)
High level	169(75.45)
Organizational size, mean (SD)	29.60(26.63)

*SD = standard deviation. The physician-level data was nested within 38 CHCs. PCPs, primary care physicians; CHCs, community health centers.

### Statistical analysis

First, descriptive statistics were used to report the basic characteristics of the sampling CHCs and PCPs. We also preliminarily tested the association between PCPs’ confidence and these basic characteristics. Second, spearman’s correlation was adopted to examine the relationship between PCPs’ confidence and the organization-level and physician-level factors. Finally, considering the physician-level data was nested within the organization-level data, we built two two-level ordinal logit models (individual at level 1; nested within the CHC at level 2) to examine the association between organization-level and physician-level factors with PCPs’ confidence in pandemic response. Our sample size was sufficient for multilevel modeling, with a mean level 1 sample size of nearly 6 PCPs and a level 2 sample size of 38 CHCs [39]. Multicollinearity analysis also suggested no existence of multicollinearity in the model. [40].

Reliability diagnosis of multilevel ordinal logit model was conducted by calculating the Intraclass Correlation Coefficient (ICC), ranging from 0 to 1, to ensure the suitability of the model for our analysis. When the value of ICC was more than 0.059, a multilevel model should be performed to analyze data. The analysis suggested that there is heterogeneity of PCPs’ pandemic response confidence among different CHCs with an ICC of 0.10 = 0.35/(0.35+ $\frac{\pi^{2}}{3}$). Considering the psychological construct may lack a meaningful zero value, the data related to organization-level predictors were mean-centered using centering at grand mean (CGM), except for the dummy variables, to facilitate interpretation of regression results. Akaike information criteria (AIC) values of the full model including both organization-level and physician-level factors (AIC: 431.55) were smaller than that of the model only including physician-level factors (AIC: 433.98), which meant full models were better fitted. Our analysis was adjusted for basic socio-demographic and organizational characteristics. We performed all statistical analyses using Stata 15.0.

## Results

Table 1 presents the basic characteristics of 224 PCPs within 38 CHCs. Over half of PCPs were female (59.82%), held undergraduate/college degrees (79.91%), and had an average age of 36.82 years, with an average working experience of 10.81 years. The 9 PCPs with high school education level were comparatively older than the average participant and based on past policy could practice in local primary care organizations after completing the required medical training and passing local health authority examinations. Approximately two-thirds of PCPs worked in government-managed CHCs (63.39%), which are part of the government sector and directly managed by the local government. The remainder worked in public hospital-managed CHCs (36.61%), which are owned by the local government but managed by the host hospital. Most PCPs worked in the CHCs with a high level of information technology functional capacity (75.45%).

Table 2 reports the distribution of organizational-level and physician-level factors. On average, PCPs reported a relatively high level of pandemic response confidence, with 97.32% of PCPs having moderate confidence and above. The average values of job skill variety, work-family conflict, perceived organizational support, and professional fulfillment at the physician level were 4.32 with 5 representing highest level of job skill variety, 16.68 with 28 representing highest level of work-family conflict, 3.54 with 7 representing highest level of organizational support, and 3.85 with 5 representing highest level of professional fulfillment, respectively. At the organization level, the mean of organization structure was 6.46 with 7 representing highest level of hierarchy of organization structure, and more than half of PCPs (75%) worked in CHCs with hierarchical-oriented culture.

**Table 2:** The correlation between organizational factors and PCPs’ confidence in pandemic response.

Characteristics	Mean(Range)	SD	1	2	3	4	5	6
1. Confidence	4.33 (1–5)	0.81	1	-	-	-	-	-
2. Skill variety	4.32 (1–5)	0.82	0.39***	1	-	-	-	-
3. Perceived organizational support	3.54 (1–7)	0.93	0.42***	0.43***	1	-	-	-
4. Work-family conflict	16.68 (4–28)	6.57	-0.14**	-0.12*	-0.39***	1	-	-
5. Professional fulfillment	3.85 (1–5)	0.85	0.38***	0.48***	0.62***	-0.37***	1	-
6. Organizational structure	6.46 (1–7)	0.74	0.09	-0.02	0.10	-0.03	0.07	1

*SD = standard deviation

*P< = 0.10

**P< = 0.05

***P< = 0.001. PCPs, primary care physicians.

Table 3 illustrates the result of two-level ordinal logit models to examine the association between organization-level and physician-level factors with PCPs’ confidence in pandemic response. In the null model, the ICC value was 0.10 suggested that the variance at the organizational level accounted for 10% of the total variance of PCPs’ confidence in pandemic response, which indicated that there was sufficient variability between CHCs to build multilevel ordinal logit models (*P*<0.05). In the full model (model 2), three physician-level factors, job skill variety (OR = 1.86, *P*<0.05), perceived organizational support (OR = 2.36, *P*<0.05), and professional fulfillment (OR = 2.26, *P*<0.05), were positively associated with PCPs’ confidence in pandemic response. One organization-level factor, hierarchical culture (OR = 3.51, *P*<0.05) was positively associated with PCPs’ pandemic response confidence. However, work-family conflict (OR = 1.05, *P*>0.05) at physician-level and organization structure (OR = 1.32, *P*>0.05) at organization-level were not associated with PCP’s pandemic response confidence.

**Table 3:** The Multilevel models examining the association of organizational factors with PCPs’ confidence in pandemic response.

Characteristics	Model 1		Model 2	
	*OR*	*95% CI*	*OR*	*95% CI*
**Physician-level**				
Perceived organizational support	2.15***	1.39, 3.32	2.36***	1.49, 3.73
Job skill variety	1.58**	1.05, 2.36	1.86***	1.23, 2.82
Work-family conflict	1.05*	0.99, 1.11	1.05	0.99, 1.11
Professional fulfillment	2.39***	1.45, 3.95	2.26***	1.36, 3.74
Gender (ref. = male)				
Female	0.81	0.45, 1.46	0.90	0.49, 1.63
Education level (ref. = high school or below)				
Undergraduate/college	0.91	0.17, 4.82	0.60	0.11, 3.24
Master or above	0.95	0.15, 6.28	0.49	0.07, 3.32
Age (ref. = <30)				
30–40	1.12	0.41, 3.04	1.11	0.41, 3.01
≥40	1.68	0.43, 6.63	1.45	0.36, 5.76
Years of working experience (ref. = <5)				
5–10	2.21	0.81, 6.07	2.29	0.83, 6.31
10–15	0.78	0.27, 2.32	0.65	0.21, 1.97
≥15	0.49	0.13, 1.87	0.52	0.13, 2.06
**Organization-level**				
Organizational structure	-	-	1.32	0.83, 2.11
Hierarchical culture (ref. = no)				
Yes	-	-	3.51**	1.09, 11.32
Ownership (ref. = government-managed)				
Public hospital-managed	-	-	3.07	1.20, 7.86
Information technology functional capacity (ref. = low or middle level)				
High level	-	-	0.59	0.23, 1.52
Organizational size	-	-	1.01	0.99, 1.03
AIC		433.55		430.03

Model 1 only included physician-level characteristics; Model 2 added organization-level characteristics. Organizational size was measured by the number of physicians in community health centers. *OR*, odd ratio; *CI*, confidence interval; AIC, Akaike information criteria.

*P< = 0.10

**P< = 0.05

***P< = 0.001.

## Discussion

Based on data from four large cities in China, we found that PCPs working in CHCs with a hierarchical culture had higher pandemic response confidence. PCPs who perceived strong organizational support and high professional commitment with varied skills and abilities also had higher pandemic response confidence. This is particularly significant given research suggesting that new and extreme contexts, such as COVID-19 pandemic, are likely to deplete confidence [41].

This study extends previous work by demonstrating the positive impact of three physician-level professional features on PCPs’ pandemic response confidence, including perceived organizational support, job skill variety, and professional fulfillment. Perceived organizational support, which reflects employee perception of the degree to which the organization appreciates their contributions and cares about their welfare [28], is an important construct that is conducive to positive psychological states in an organizational setting [42, 43]. When perceived organizational support is high, employees are likely to expect that they will have sufficient resources and support to effectively perform the tasks associated with their role, including challenging and complex tasks, which likely contributes to enhanced confidence [44]. In CHCs with supportive working environments, such as adequate personal protective equipment, financial subsidy, COVID-19 relevant training, PCPs are more likely to perceive that they are able to effectively respond to challenges associated with the pandemic, reflecting higher pandemic response confidence.

Job skill variety, as a core job characteristic fostering positive psychological states, is defined as the extent to which a job requires various activities in its execution [45]. When employees engage in a job requiring them to undertake challenges or stretch their skills and abilities, they may experience three critical psychological states (job meaningfulness, a sense of responsibility, and knowledge of work outcome), which lead to favorable outcomes including increased internal work motivation, work performance, job satisfaction, and confidence [41, 46, 47]. As China has lifted strict zero-Covid policy, PCPs are experiencing increasing heavy workloads to manage COVID-19 patients while providing regular care. PCPs with varied skills and abilities in this challenging work context are more likely to perceive that their job is meaningful, that they are responsible for impactful work and be better able to see the outcome of their job, all of which contribute to higher pandemic response confidence.

Professional fulfillment, which is defined as “the degree of intrinsic positive rewards that an individual derives from his or her work, such as satisfaction, happiness, and meaningfulness”, has been linked to a variety of physicians’ psychological states, as well as behavior and performance [30, 48–50]. For example, professional fulfillment is inversely related to burnout during the pandemic among surgeons [50]. Physicians with high professional fulfillment are more likely to work efficiently and develop strong collegial networks. Further, professionally fulfilled professionals tend to develop and deploy high level work skills, and have a deep intrinsic motivation to provide optimal care [49, 51]. Thus, PCPs with a high level of professional fulfillment are more likely to devote themselves to responding effectively to the pandemic, and continue to make significant personal investments in pandemic relevant learning and training as well as exhibit high levels of engagement [48, 52]. Therefore, PCPs with high professional fulfillment have high pandemic response confidence.

However, our data revealed that the association between work-family conflict and PCPs’ confidence in pandemic response was not statistically significant. Role theory posits that inter-role conflict exists when individuals face difficulties in their efforts to successfully perform the requirements of different roles due to constrained resources or role incompatibility [53]. Work-family conflict is a specific type of inter-role conflict in which greater participation in a work role hinders the capacity to meet the demands of the family role [54]. A substantial body of evidence suggests that tensions between work and family roles can lead to decrements in the psychological well-being of physicians, including burnout, depression, job dissatisfaction, and high work stress, which may affect perceived confidence in coping with challenging context [41, 55–57]. However, a strong sense of responsibility, collective action, and family support among healthcare professionals in China has been reported during the pandemic [58, 59]. This context may alleviate the adverse effect of work-family conflict, which might explain our findings of a non-significant association, suggesting an avenue for further study [58–62]. In addition, our multilevel modelling suggested that the link between confidence in pandemic response and age and working experience was not statistically significant. Further study might explore the potential reasons explaining this result.

Hierarchical culture reflects control, standardization, and stability [25]. This study revealed the positive impact of hierarchical culture on PCPs’ pandemic response confidence. As healthcare provision during the pandemic is characterized by rapid change, complexity and uncertainty, the positive impact of hierarchical culture may be specific to this context. In China, medical institutions, including CHCs, are required by the Chinese government to strictly implement national technical guidelines for preventing and controlling COVID-19 [63]. Medical institutions follow the national guidelines and reinforce the operational norms of specific work content related to the pandemic response. Supported by the hierarchical system and structured guidelines, frontline healthcare workers are supported to rapidly shift their routine work content to pandemic relevant activities. An organizational culture that supports the implementation of national structured guidelines provides a clear sense of PCPs’ responsibilities and required actions, which could increase their confidence in pandemic response.

Our study should be considered in light of its limitations. First, this study examined the association rather than the causality between the organizational factors and PCP’ confidence due to the cross-sectional design. We suggest that future work could leverage longitudinal research to examine causality in our supported relationships. Second, 224 PCPs from 38 CHCs in four cities were selected in the study. As we conducted the data collection in person during the pandemic in China, we collected data from a sample size sufficient for statistical modeling while also considering the representativeness of the sample. Based on the results and experiences for this small-scale study, further study with a large sample size may be useful in validating our results in different contexts. Third, this study examined the direct effect of organizational factors on PCPs’ pandemic response confidence, without considering the interaction between the various organization-level and physician-level factors. Further research should be conducted to examine the interaction effect of these factors. Fourth, only one item was used to measure PCPs’ confidence in the pandemic response and we did not measure their knowledge, skills and actual involvement in the pandemic response. It is possible that some study participants may be highly competent, but have low confidence in pandemic response due to organizational and system barriers. More comprehensive measures of pandemic response confidence are needed to address this limitation.

## Conclusions

As the first-line health care provider in the community, PCPs play a significant role in pandemic response. Building their pandemic response confidence is likely to not only improve their wellbeing but also improve their work performance and maintain the sustainability of the pandemic response. This study not only increases our understanding of the influence of organizational context on PCPs’ COVID-19 response confidence, but also points out potential management levers for front-line primary care managers to build primary care pandemic response capacity by, for example, enhancing the skill training for personal ability development and emergency response, valuing the contributions of practitioners and care about their well-being, and clarifying job responsibilities.

## Supporting information

S1 Checklist (DOCX)

S1 Data (XLSX)
